# Supplementary material for: CoPoP liposomes displaying stabilized clade C HIV-1 Env elicit tier 2 multiclade neutralization in rabbits
Source: Nat Commun. 2024 Apr 11;15:3128. doi: 10.1038/s41467-024-47492-1 (PMC11009251; doi:10.1038/s41467-024-47492-1)
Supplement: Supplementary file 1 — Supplementary Information [file 41467_2024_47492_MOESM1_ESM.pdf]

Supplementary figure 1

Soluble His-tagged Env sequences	
ConC	MRVRGILRNWQQWWIWGILGFWMLMICNVVGNLWVTVYYGVPVWKEAKTTLFCASDAKAYEKEVHNVWATHACVPTDPNPQEMVLENVTENFNMWK<br>NDMVDQMHEDIISLWDQSLKPCVKLTPLCVTLNCTNVNVTEMKNCSFNTTTEIRDKKQKEYALFYRLDIVPLNENSSEYRLINCNTSTITQICPKV<br>SFDPIPIHYCAPAGYAILKCNNKTFNGTGPCNNVSTVQCTHGIKPVVSTQLLLNGSLAEEEIIIRSENLTNNVKTIIVHLNESVEIVCTRPNNNTR<br>KSIRIGPGQTFYATGDIIGDIRQAHCNISEAKWNKTLQRVKKKLKEHFPNKTIKFQPSSGGDLEITTHSFNCRGEFFYCNTSKLFNSTYNNTTSNS<br>TITLPCRIKQIINMWQEVGRAMYAPPIAGNITCKSNITGLLLTRDGGNNNNNTETFRPGGGDMRDNWRSELYKYKVVEIKPLGIAPTKCKRRVVER<br>RRRRRAVGIGAVFLGFLGAAGSTMGAASNTLTVQARQLLSGIVQQQSNLLRAPEAQQHMLQLTVWGFKQLQARVLAIERYLEVQQLLGIWGCSGKL<br>ICCTAVPWNSSWSNKSQEDIWDNMTWMQWDREISNYTDTIYRLLEESQFQQEINEKDLLALDGGGGSLPETGGGSGGGGSGHHHHHHHH
sC4	MRVRGMLRNWQQWWIWSSLGFWMLMIYSVMGNLWVTVYYGVPVWKDAKTTLFCASDAKAYEKEVHNVWATHACVPTDPNPQEIVLGNVTENFNMWK<br>NDMVDQMHEDIISLWDQSLKPCVKLTPLCVTLNCRNVRNVEMKNCSFNATTVVRDRKQKVHALFYRLDIVPLDENNSSYRLINCNTSACTQICPKV<br>SFDPIPIHYCAPAGYAILKCNNKTFNGTGPCNNVSTVQCTHGIKPVVSTQLLLNGSLAEEEIIIRSENLTNNAKTIIVHLNETVNINCTRPNNNTR<br>KSIRIGPGQTFYATGDIIGDIRQAHCNLSRDGWNKTLQGVKKKLAEHFPNKTIKFAPHSGGDLEITTHSFNCRGEFFYCNTSNLFNESNIERNDSI<br>ITLPCRIKQIINMWQEVGRCMYAPPIAGNITCRSNITGLLLTRDGGSNNNDTETFRPGGGDMRNNWRSELYKYKVVEVKPLGVAPTECKRRVVERR<br>RRRRAVGIGAVFLGILGAAGSTMGAASNTLTVQARQLLSGIVQQQSNLPRAPEAQQHMLQLTVWGIKQLQTRVLAIERYLEDQQLLGLWGCSGKLI<br>CCTAVPWNTSWSNKSQTDIWDNMTWMQWDKEIGNYTGEIYRLLEESQFQQEIGSGGGGGSLPETGGGSGGGGSGHHHHHHHH
C97ZA	MRVRGILRNWQQWWIWGILGFWMLMICNVVGNLWVTVYYGVPVWKDAKTTLFCASDAKAYDREVHNVWATHACVPTDPNPQEIVLENVTENFNMWK<br>NDMVDQMHEDIISLWDQSLKPCVKLTPLCVTLHCTNATFKNNVTNDMNKEIRNCSFNTTTEIRDKKQKVYALFYRLDIVQLKENRNNSNNSEYRLI<br>NCNTSTCTQICPKVTFDPIPIHYCAPAGYAILKCNNKTFNGTGPCNNVSTVQCTHGIKPVVSTQLLLNGSLAEKEIIIRSENLTDNVKTIIVHLNK<br>SVEINCTRPNNNTRKSIRIGPGQTFYATGDIIGDIRQAYCNISGSKWNETLKRVKEKLREHFNNNKTIKFAPSSGGDLEITTHSFNCRGEFFYCNT<br>TRLFNNNATENETITLPCRIKQIINMWQEVGRCMYAPPIAGNITCKSNITGLLLTRDGGEDNKTEEIFRPGGGNMKDNWRSELYKYKVVEIKPLGI<br>APTKCKRRNVTRRRRRRAVGIGAVFLGFLGAAGSTMGAASNTLTVQARQLLSGIVQQQSNLPRAPEAQQHMLKLTVWGIKQLQARVLAIERYLEVQ<br>QLLGIWGCSGKLICCTNVPWNSSWSNKSQTDIWNNMTWMEWDREISNYTDTIYRLLEDSQFQQEINEVDLLANDGGGGSLPETGGGSGGGGSGHHH<br>HHHHH
ConB	MRVKGIRKNYQHLWRWGTMLLGMLMICSAAEKLWVTVYYGVPVWKEATTTLFCASDAKAYDTEVHNVWATHACVPTDPNPQEVVLENVTENFNMWK<br>NNMVEQMHEDIISLWDQSLKPCVKLTPLCVTLNCTDLNNNTTNNNSSSEKMEKGEIKNCSFNITTSIRDKVQKEYALFYKLDVVPIDNNNTSYRLI<br>SCNTSVITQACPKVSFEPIPIHYCAPAGFAILKCNDKKFNGTGPCTNVSTVQCTHGIRPVVSTQLLLNGSLAEEEVVIRSENFTDNAKTIIVQLNE<br>SVEINCTRPNNNTRKSIHIGPGRAFYATGDIIGDIRQAHCNISRTKWNNTLKQIVKKLREQFGNKTIVFNQSSGGDPEIVMHSFNCGGEFFYCNTT<br>QLFNSTWNSNGTWNNTTGNDTITLPCRIKQIINMWQEVGKAMYAPPIRGQIRCSSNITGLLLTRDGGNNNNNTTETFRPGGGDMRDNWRSELYKYK<br>VVKIEPLGVAPTKCKRRVVQRRRRRRAVGIGAMFLGFLGAAGSTMGAASITLTVQARQLLSGIVQQQNNLLRAPEAQQHLLQLTVWGIKQLQARVL<br>AVERYLKDQQLLGIWGCSGKLICCTAVPWNTSWSNKSLDEIWDNMTWMQWEREIDNYTGLIYTLIEESQNQQEKNEQELLELDAAALPETGGGSDY<br>KDDDDKPGGGGSGGGGSGGGGSGGGGSGGGGSGGGGSGGGGSHHHHHH
BG505	MRVMGIQRNCQHLFRWGTMILGMIIICSAAENLWVTVYYGVPVWKDAETTLFCASDAKAYETEKHNVWATHACVPTDPNPQEIHLENVTEEFNMWK<br>NNMVEQMHTDIISLWDQSLKPCVKLTPLCVTLQCTNVTNNITDDMRGELKNCSFNMTTELRDKKQKVYSLFYRLDVVQINENQGNRSNNSNKEYRL<br>INCNTSACTQACPKVSFEPIPIHYCAPAGFAILKCKDKKFNGTGPCPSVSTVQCTHGIKPVVSTQLLLNGSLAEEEVMIRSENITNNAKNILVQFN<br>TPVQINCTRPNNNTRKSIRIGPGQAFYATGDIIGDIRQAHCNVSKATWNETLGKVVKQLRKHFGNNTIIRFANSSGGDLEVTTHSFNCGGEFFYCN<br>TSGLFNSTWISNTSVQGSNSTGSNDSITLPCRIKQIINMWQRIGQCMYAPPIQGVIRCVSNITGLILTRDGGSTNSTTETFRPGGGDMRDNWRSEL<br>YKYKVVKIEPLGVAPTRCKRRVVGRRRRRRAVGIGAVFLGFLGAAGSTMGAASNTLTVQARNLLSGIVQQQSNLPRAPEAQQHLLKLTVWGIKQLQ<br>ARVLAVERYLEVQQLLGIWGCSGKLICCTNVPWNSSWSNRNLSEIWDNMTWLQWDKEISNYTQIIYGLLEESQFQQEINEVDLLALDGGGGSLPET<br>GGGSGGGGSGHHHHHHHH

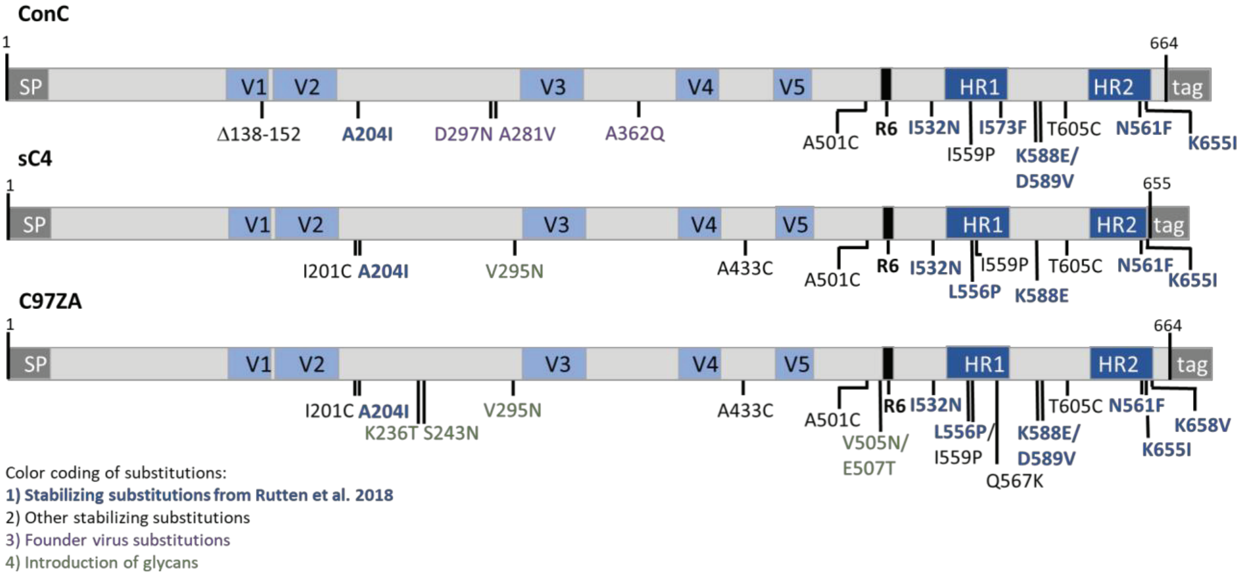

Supplementary figure 1:
Sequence of soluble His-tagged RnS clade C HIV-1 Env antigens, ConC, sC4, and C97ZA, and of control proteins BG505 and ConB. Including a schematic representation of introduced stabilizing mutations in the clade C antigens.

Supplementary figure 2

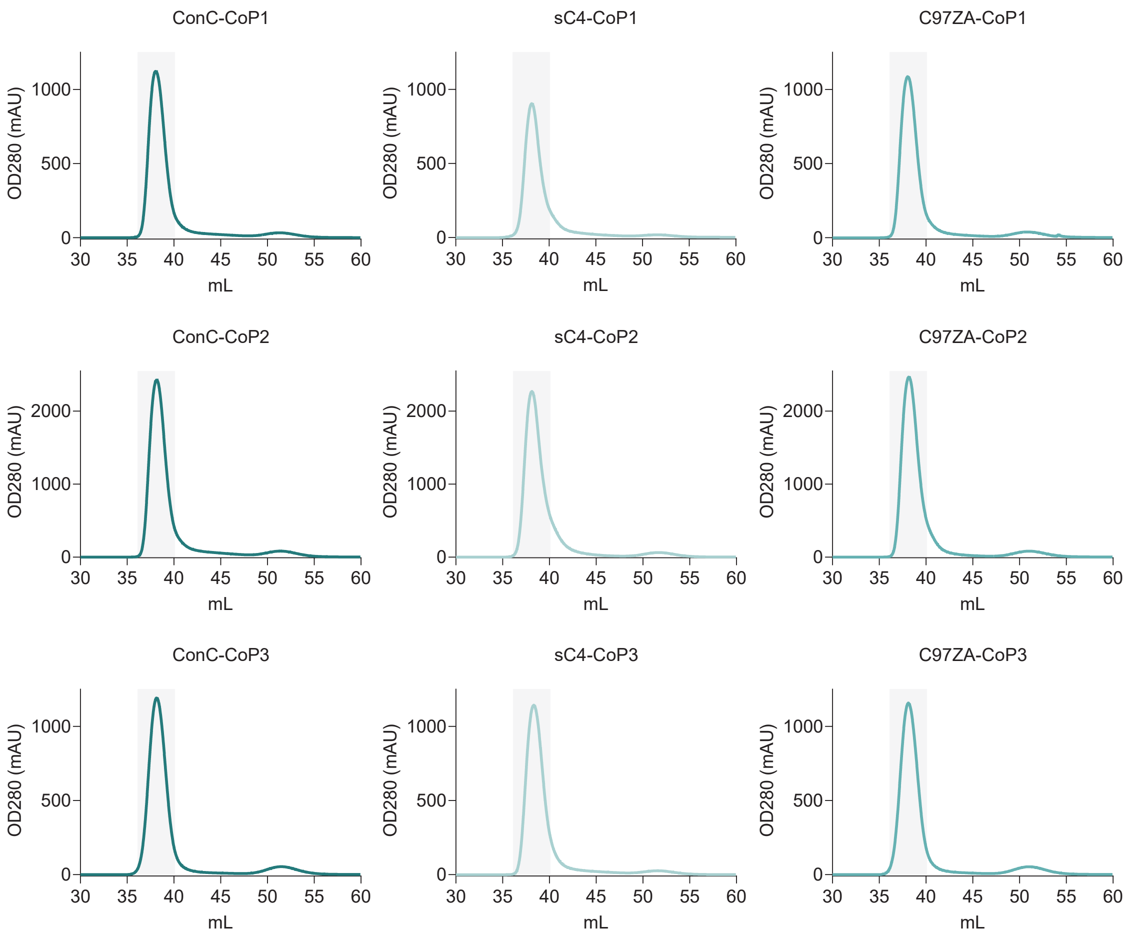

Supplementary figure 2:
Size Exclusion Chromatography (SEC) purification profile of Env-CoPoP liposomes. The collected Env-CoPoP liposome fraction is indicated by the shaded area between 35-40 mL retention time. The unbound Env fraction elutes at approximately 50-55 mL. Source data are provided as a Source Data file.

Supplementary figure 3

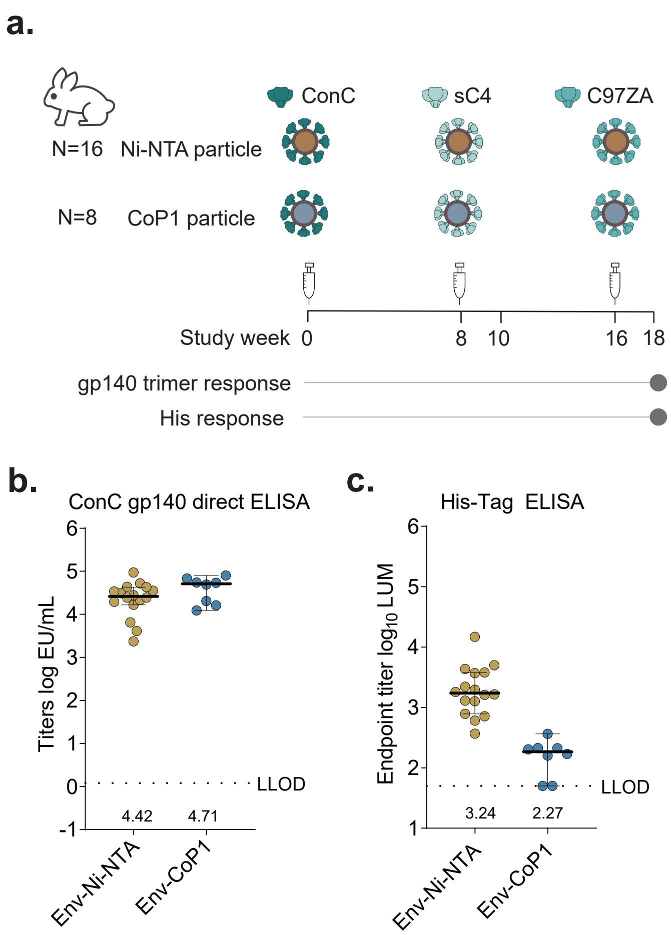

Supplementary figure 3:

a, Schematic of a prior study where Ni-NTA liposomes were compared to CoP1 liposomes. Direct ELISA titers of Env-CoP1 (N=8) and Env-Ni-NTA (N=16) immunized animals the study in panel a comparing the CoP1 particles to Ni-NTA, tested against ConC gp140 trimer (**b**) and His-tag (**c**). Bars in b and c represent median with 95% confidence interval (CI) and numbers at the bottom of the graphs denote median values. Dotted lines in b and c show lower limit of detection (LLOD). Source data are provided as a Source Data file.

Supplementary figure 4

a.

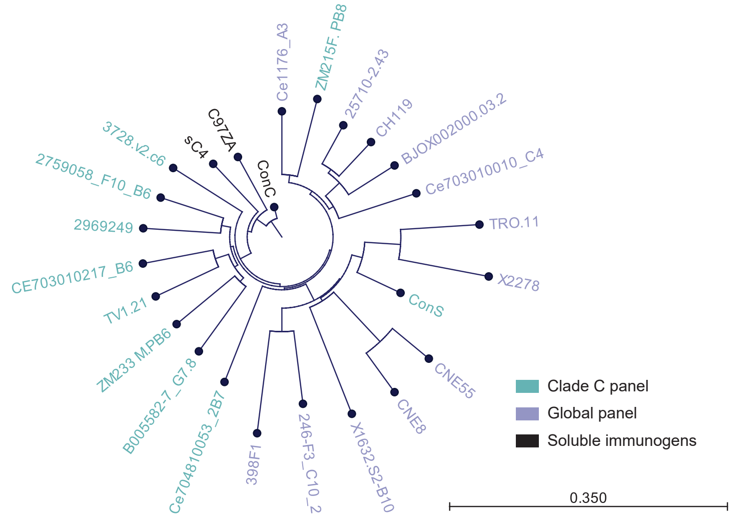

b.

	Ce704810053_2B7	ZM215F. PB8	2759058_F10_B6	2969249	3728.v2.c6	Ce703010010_C4	BJOX002000.03.2	CH119	25710-2.43	Ce1176_A3	ConS	ConC	C97ZA	sC4	Ce703010217_B6	TV1.21	ZM233 M.PB6	B005582-7_G7.8	398F1	246-F3_C10_2	X1632.S2-B10	CNE8	CNE55	X2278	TRO.11
Ce704810053_2B7		0.22	0.21	0.23	0.21	0.22	0.22	0.22	0.21	0.20	0.21	0.16	0.20	0.22	0.23	0.24	0.22	0.22	0.30	0.25	0.26	0.27	0.27	0.30	0.28
ZM215F. PB8	76.30		0.18	0.18	0.20	0.20	0.20	0.19	0.17	0.16	0.21	0.16	0.20	0.21	0.20	0.21	0.22	0.22	0.27	0.25	0.27	0.25	0.25	0.28	0.27
2759058_F10_B6	76.24	78.61		0.16	0.18	0.20	0.19	0.19	0.19	0.18	0.19	0.14	0.19	0.18	0.19	0.19	0.18	0.19	0.26	0.22	0.24	0.24	0.24	0.30	0.25
2969249	75.31	79.15	83.04		0.20	0.22	0.23	0.22	0.21	0.20	0.20	0.15	0.17	0.19	0.21	0.20	0.18	0.19	0.27	0.22	0.25	0.25	0.26	0.30	0.27
3728.v2.c6	78.39	79.26	79.25	78.06		0.21	0.18	0.19	0.18	0.20	0.21	0.15	0.18	0.18	0.20	0.21	0.19	0.21	0.27	0.22	0.26	0.27	0.26	0.28	0.26
Ce703010010_C4	77.27	78.59	78.90	77.52	78.32		0.18	0.16	0.18	0.18	0.20	0.14	0.19	0.18	0.20	0.21	0.20	0.21	0.27	0.24	0.28	0.27	0.27	0.28	0.28
BJOX002000.03.2	76.88	78.03	78.62	75.59	79.65	79.75		0.12	0.12	0.17	0.18	0.14	0.18	0.18	0.21	0.20	0.19	0.19	0.24	0.22	0.25	0.26	0.26	0.27	0.25
CH119	76.08	77.59	80.06	77.10	78.91	80.72	86.91		0.10	0.17	0.16	0.14	0.18	0.17	0.20	0.20	0.18	0.20	0.25	0.22	0.25	0.25	0.25	0.27	0.27
25710-2.43	76.83	80.76	79.69	77.97	80.31	80.94	85.92	87.89		0.13	0.17	0.14	0.19	0.17	0.20	0.20	0.19	0.19	0.25	0.21	0.25	0.25	0.24	0.27	0.26
Ce1176_A3	79.84	82.37	79.15	78.16	81.28	80.06	81.36	81.69	84.28		0.17	0.13	0.18	0.18	0.17	0.18	0.20	0.20	0.25	0.23	0.23	0.26	0.26	0.27	0.24
ConS	78.53	77.74	77.88	76.97	79.37	79.25	79.66	81.18	81.76	82.15		0.13	0.18	0.18	0.21	0.19	0.20	0.19	0.19	0.19	0.20	0.20	0.21	0.19	0.19
ConC	80.63	81.74	81.55	80.16	83.49	83.12	81.55	81.00	82.59	83.94	83.94		0.10	0.11	0.15	0.14	0.15	0.15	0.23	0.19	0.22	0.22	0.22	0.25	0.23
C97ZA	76.55	78.24	77.62	78.73	79.62	79.50	77.57	77.93	78.72	79.15	80.47	87.96		0.15	0.21	0.20	0.19	0.18	0.27	0.22	0.26	0.23	0.25	0.27	0.27
sC4	73.87	76.49	77.76	75.12	78.82	78.29	76.61	76.90	78.74	77.73	78.04	87.56	82.96		0.20	0.18	0.19	0.20	0.27	0.22	0.26	0.27	0.25	0.28	0.27
Ce703010217_B6	76.21	76.78	77.78	75.43	77.52	77.93	77.12	77.05	77.78	80.72	77.86	80.88	76.68	75.83		0.16	0.19	0.21	0.27	0.23	0.24	0.26	0.26	0.27	0.26
TV1.21	77.26	76.44	77.30	77.42	78.00	77.31	77.31	77.49	77.57	80.84	79.84	81.68	76.11	76.54	80.83		0.18	0.19	0.28	0.23	0.26	0.25	0.25	0.29	0.28
ZM233 M.PB6	76.66	77.54	79.43	78.37	80.74	79.74	78.27	78.44	79.52	79.30	79.30	84.55	80.29	79.00	78.92	79.16		0.18	0.28	0.24	0.27	0.28	0.26	0.27	0.26
B005582-7_G7.8	77.81	76.67	79.47	79.94	78.39	78.30	78.35	77.90	78.50	79.43	78.66	81.96	79.47	76.54	76.55	79.32	81.11		0.28	0.22	0.26	0.27	0.26	0.28	0.27
398F1	70.23	72.66	72.90	71.25	74.01	72.61	74.03	73.84	74.92	75.43	80.03	76.40	74.17	73.05	71.65	71.85	74.40	72.77		0.20	0.27	0.28	0.29	0.29	0.29
246-F3_C10_2	74.41	74.17	74.88	73.73	77.41	74.80	75.55	75.54	77.43	77.01	80.13	79.62	77.85	75.80	76.06	75.58	77.00	75.74	79.87		0.27	0.25	0.26	0.28	0.28
X1632.S2-B10	73.76	73.22	73.76	73.37	74.80	72.50	73.07	72.77	74.30	75.78	78.87	76.86	74.21	72.63	73.49	73.81	74.09	74.06	74.92	74.10		0.26	0.27	0.30	0.28
CNE8	73.58	74.72	74.45	74.84	73.93	73.19	73.86	73.07	74.18	74.84	77.78	77.28	75.43	71.70	72.07	74.38	73.48	75.40	72.76	73.87	74.37		0.11	0.30	0.27
CNE55	73.66	74.20	74.10	73.47	74.84	73.49	72.30	72.29	73.87	74.37	77.08	77.37	74.37	73.21	73.11	74.34	75.08	74.92	71.50	73.93	73.27	87.82		0.29	0.28
X2278	71.72	71.84	70.30	70.87	73.30	72.50	71.85	71.65	72.40	74.37	78.78	74.53	72.07	70.44	71.94	72.40	73.89	73.97	72.21	72.39	71.72	72.97	72.70		0.20
TRO.11	72.95	70.96	71.95	70.71	73.56	71.76	72.63	71.43	72.50	74.81	79.63	75.04	73.41	71.61	73.42	73.43	74.33	72.62	72.90	73.75	73.25	71.65	71.52	78.43	

Upper comparison: Distance ; Lower comparison: % identity

Min — Max

c.

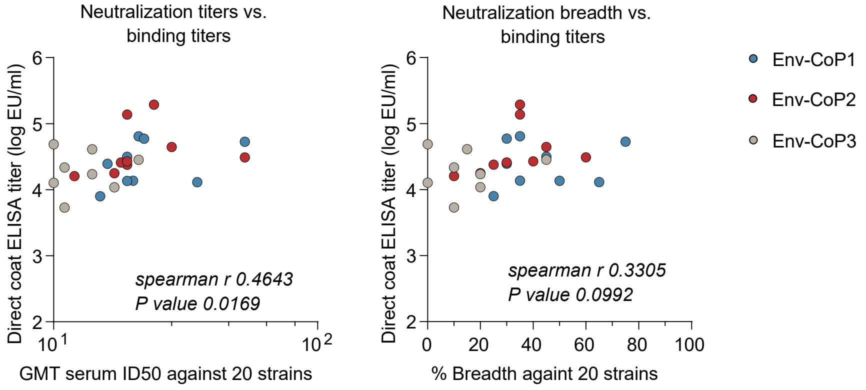

Supplementary figure 4:

a, Circular phylogenetic tree of the soluble immunogen strains (n=3) and pseudovirus strains (n=22) used in the study. **b**, Distance matrix table of the HIV-1 strains shown in panel a. **c**, Spearman correlation of geometric mean titer (GMT) neutralization and % breadth vs. gp140 trimer binding at week 18 per rabbit. Colors depict the different groups as indicated. Source data are provided as a Source Data file.

Supplementary figure 5

a.

Membrane-bound Env sequences	
ConC	MRVRGILRNWQQWWIWGILGFWMLMICNVVGNLWVTVYYGVPVWKEAKTTLFCASDAKAYEKEVHNVWATHACVPTDPNPQEMVLENVTENFNMWK<br>NDMVDQMHEDIISLWDQSLKPCVKLTPLCVTLNCTNVNVTNTNNNNMKEEMKNCSFNTTTEIRDKKQKEYALFYRLDIVPLNENSSEYRLINCNTS<br>TITQICPKVSFDPIPIHYCAPAGYAILKCNNKTFNGTGPCNNVSTVQCTHGIKPVVSTQLLLNGSLAEEEIIIRSENLTDNAKTIIVHLNESVEIV<br>CTRPNNMTVKSIRIGPGQTFYALGDIIGDIRQAHCNISEAKWNKTLQRVKKKLKEHFPNKTIKFAPSSGGDLEITTHSFNCRGEFFYCNTSKLFNS<br>TYNNTTSNSTITLPCRIKQIINMWQEVGRAMYAPPIAGNITCKSNITGLLLTRDGGNNNNNTETFRPGGGDMRDNWRSELYKYKVVEIKPLGIAPT<br>KAKRRVVEGGGGSGGGGSAVGIGAVFLGFLGAAGSTMGAASNTLTVQARQLLSGIVQQQSNLLRAPEAQQHMLQLTVWGFKQLQARVLAIERYLEV<br>QQLLGIWGCSGKLICTTAVPWNSSWSNKSQEDIWDNMTWMQWDREISNYTDTIYRLLEESQFQQEINEKDLLALDSWNNLWNWFDITNWLWYIKIF<br>IMIVGGLIGLRIIFAVLSIVNRVRQGY
sC4	MRVRGMLRNWQQWWIWSSLGFWMLMIYSVMGNLWVTVYYGVPVWKDAKTTLFCASDAKAYEKEVHNVWATHACVPTDPNPQEIVLGNVTENFNMWK<br>NDMVDQMHEDIISLWDQSLKPCVKLTPLCVTLNCRNVRNVEMKNCSFNATTVVRDRKQKVHALFYRLDIVPLDENNSSYRLINCNTSAITQICPKV<br>SFDPIPIHYCAPAGYAILKCNNKTFNGTGPCNNVSTVQCTHGIKPVVSTQLLLNGSLAEEEIIIRSENLTNNAKTIIVHLNETVNITCTRPNNMTV<br>KSIRIGPGQTFYALGDIIGDIRQAHCNLSRDGWNKTLQGVKKKLAEHFPNKTIKFAPHSGGDLEITTHSFNCRGEFFYCNTSNLFNESNIERNDSI<br>ITLPCRIKQIINMWQEVGRAMYAPPIAGNITCRSNITGLLLTRDGGSNNNDTETFRPGGGDMRNNWRSELYKYKVVEVKPLGVAPTEAKRRVVEGG<br>GGSGGGGSAVGIGAVFLGILGAAGSTMGAASNTLTVQARQLLSGIVQQQSNLPRAPEAQQHMLQLTVWGIKQLQTRVLAIERYLEVQQLLGLWGCS<br>GKLICTTAVPWNTSWSNKSQTDIWDNMTWMQWDKEIGNYTGEIYRLLEESQFQQEINEKDLLALDSWNNLWNWFDITNWLWYIKIFIMIVGGLIGL<br>RIIFAVLSIVNRVRQGY
C97ZA	MRVRGILRNWQQWWIWGILGFWMLMICNVVGNLWVTVYYGVPVWKDAKTTLFCASDAKAYDREVHNVWATHACVPTDPNPQEIVLENVTENFNMWK<br>NDMVDQMHEDIISLWDQSLKPCVKLTPLCVTLHCTNATFKNNVTNDMNKEIRNCSFNTTTEIRDKKQKVYALFYRLDIVQLKENRNNSNNSEYRLI<br>NCNTSTITQICPKVTFDPIPIHYCAPAGYAILKCNNKTFNGTGPCNNVSTVQCTHGIKPVVSTQLLLNGSLAEKEIIIRSENLTDNVKTIIVHLNK<br>SVEIVCTRPNNMTVKSIRIGPGQTFYALGDIIGDIRQAYCNISGSKWNETLKRVKEKLREHFNNNKTIKFAPSSGGDLEITTHSFNCRGEFFYCNT<br>TRLFNNNATENETITLPCRIKQIINMWQEVGRAMYAPPIAGNITCKSNITGLLLTRDGGEDNKTEEIFRPGGGNMKDNWRSELYKYKVVEIKPLGI<br>APTKAKRRVVEGGGGGSGGGGSAVGIGAVFLGFLGAAGSTMGAASNTLTVQARQLLSGIVQQQSNLPRAPEAQQHMLKLTVWGIKQLQARVLAIER<br>YLEVQQLLGIWGCSGKLICTTNVPWNSSWSNKSQTDIWNNMTWMEWDREISNYTDTIYRLLEDSQFQQEINEKDLLALDSWNNLWNWFDITNWLWY<br>IKIFIMIVGGLIGLRIIFAVLSIVNRVRQGY

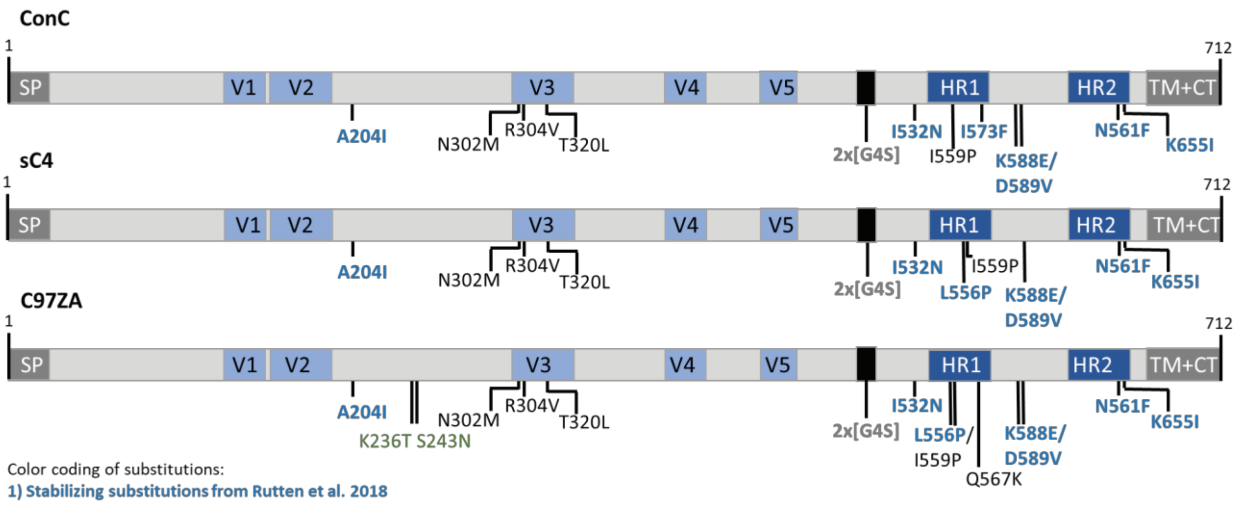

Color coding of substitutions:
1) **Stabilizing substitutions from Rutten et al. 2018**
2) Other stabilizing substitutions
3) Introduction of glycans

b.

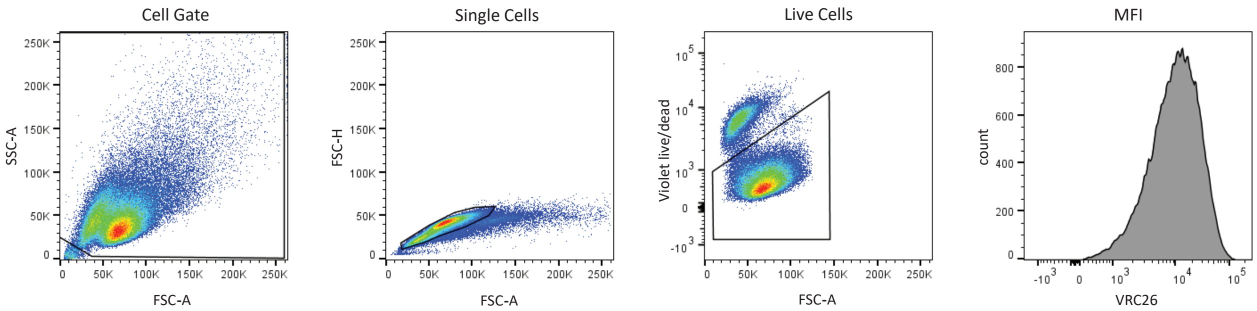

Supplementary figure 5:
a, Sequence of membrane-bound RnS clade C HIV-1 Env antigens, ConC, sC4, and C97ZA used in trivalent cocktail Ad26.RnS.Env. Including a schematic representation of introduced stabilizing mutations in the clade C antigens. **b**, Flow cytometry gating strategy, selecting the A549, single, live cell population. The median fluorescence intensity (MFI) of the Env-AF647 signal derived from the fourth gating panel "MFI" of each sample is reported in Figure 4a. Example shown: VRC26 binding to Ad26.RnS.Env-transduced cells.

Supplementary Figure 6

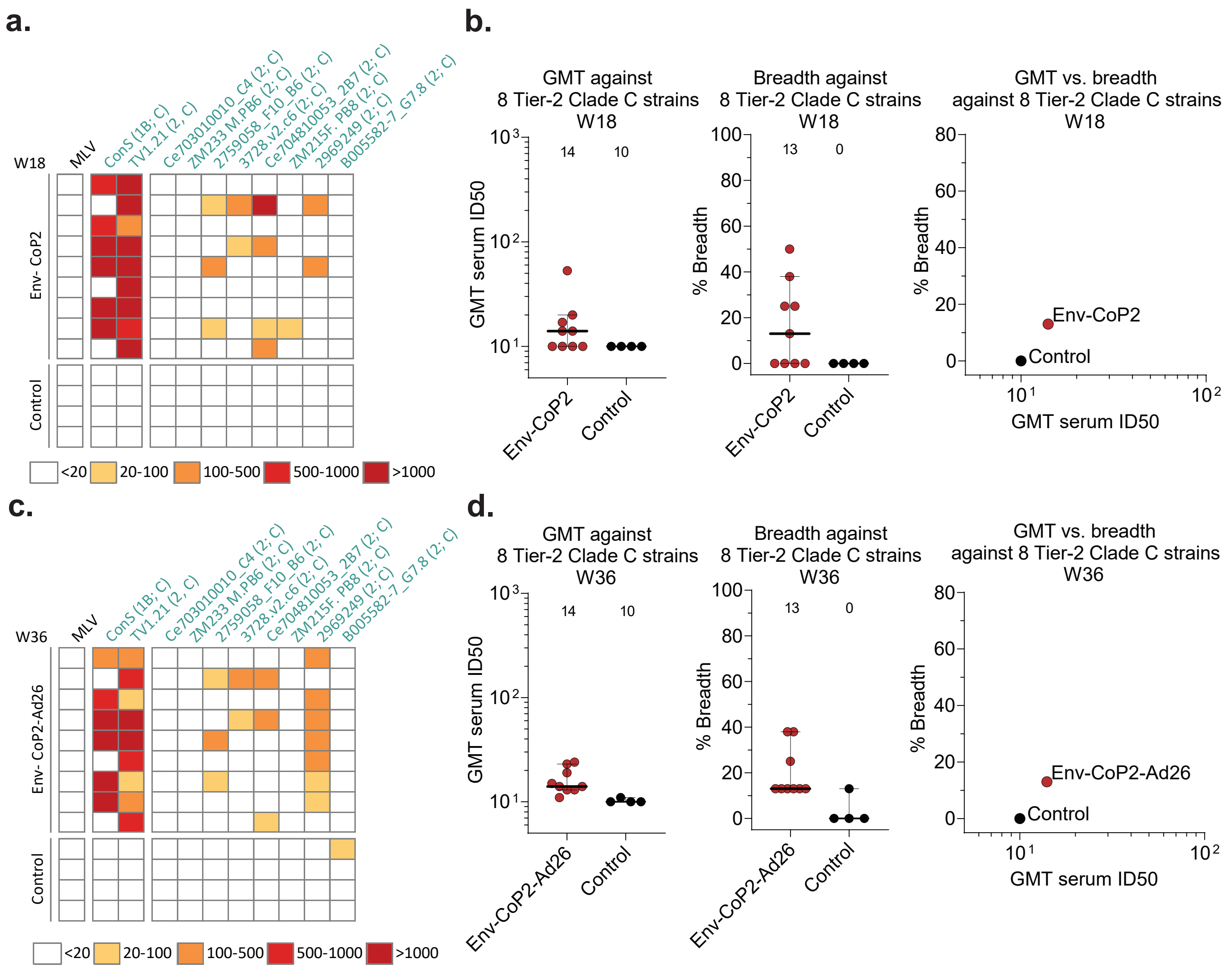

Supplementary figure 6:

a and **c**, Color gradient map depicting the serum neutralizing activity of sera from the Env-CoP2 (N=9) and control (N=4) groups derived two weeks after completion of three-dose Env-CoPoP immunizations (week 18) and post Ad26 boost (week 36) against Murine Leukemia Virus (MLV) and the n=10 strain clade C panel. **b** and **d**, Left: Scatter plot showing geometric mean titer (GMT) of serum neutralization against the n=8 tier 2 clade C strains. Right: Scatter plot showing neutralization breadth per rabbit, calculated as the % of strains neutralized out of the n=8 tested tier 2 strains. Dot plot showing GMT neutralization vs. median % breadth for all groups in the study. Values below the neutralization assay lower limit of detection (LLOD) were set to ½ the LLOD of 10 serum ID50 for plotting and analysis. Bars represent median with 95% confidece interval (CI) and numbers at the top of the graphs denote median values. Source data are provided as a Source Data file.

Supplementary Table 1

Figure	Parameter	Groups	Significant*	P value	Test
Fig. 2b	ConC direct ELISA	Env-CoP1 vs. Env-CoP2	No	0.4697	One-way ANOVA with Tukey's multiple comparison
Fig. 2b	ConC direct ELISA	Env-CoP1 vs. Env-CoP3	No	0.783	One-way ANOVA with Tukey's multiple comparison
Fig. 2b	ConC direct ELISA	Env-CoP2 vs. Env-CoP3	No	0.1834	One-way ANOVA with Tukey's multiple comparison
Fig. 2c	ConC capture ELISA	Env-CoP1 vs. Env-CoP2	No	0.9996	One-way ANOVA with Tukey's multiple comparison
Fig. 2c	ConC capture ELISA	Env-CoP1 vs. Env-CoP3	No	>0.9999	One-way ANOVA with Tukey's multiple comparison
Fig. 2c	ConC capture ELISA	Env-CoP2 vs. Env-CoP3	No	0.9995	One-way ANOVA with Tukey's multiple comparison
Fig. 2d	His-Tag ELISA	Env-CoP1 vs. Env-CoP2	No	0.993	One-way ANOVA with Tukey's multiple comparison
Fig. 2d	His-Tag ELISA	Env-CoP1 vs. Env-CoP3	No	0.8336	One-way ANOVA with Tukey's multiple comparison
Fig. 2d	His-Tag ELISA	Env-CoP2 vs. Env-CoP3	No	0.7736	One-way ANOVA with Tukey's multiple comparison
Fig. S3b	ConC direct ELISA	Env-CoP1 vs. Env-Ni-NTA	No	0.1895	Two-tailed Unpaired t test
Fig. S3c	His-Tag ELISA	Env-CoP1 vs. Env-Ni-NTA	Yes	<0.0001	Two-tailed Unpaired t test

Figure	Parameter	Groups	Significant*	P value	Test
Fig. 3b	GMT serum ID50 VNA	Env-CoP1 vs. Env-CoP2	No	>0.9999	Kruskal-Wallis test with Dunn's multiple comparisons
Fig. 3b	GMT serum ID50 VNA	Env-CoP1 vs. Env-CoP3	Yes	0.0125	Kruskal-Wallis test with Dunn's multiple comparisons
Fig. 3b	GMT serum ID50 VNA	Env-CoP2 vs. Env-CoP3	Yes	0.0369	Kruskal-Wallis test with Dunn's multiple comparisons
Fig. 3b	% Breadth VNA	Env-CoP1 vs. Env-CoP2	No	0.9652	Kruskal-Wallis test with Dunn's multiple comparisons
Fig. 3b	% Breadth VNA	Env-CoP1 vs. Env-CoP3	Yes	0.0055	Kruskal-Wallis test with Dunn's multiple comparisons
Fig. 3b	% Breadth VNA	Env-CoP2 vs. Env-CoP3	No	0.0934	Kruskal-Wallis test with Dunn's multiple comparisons

Figure	Parameter	Groups	Significant*	P value	Test
Fig. 4c	ConC direct ELISA	Env-CoP2; W18 vs. W34	Yes	<0.0001	Two-tailed paired t test
Fig. 4c	ConC direct ELISA	Env-CoP2; W18 vs. W36	No	0.0821	Two-tailed paired t test
Fig. 4c	ConC direct ELISA	Env-CoP2; W34 vs. W36	Yes	<0.0001	Two-tailed paired t test
Fig. 4d	ConC capture ELISA	Env-CoP2; W18 vs. W34	Yes	<0.0001	Two-tailed paired t test
Fig. 4d	ConC capture ELISA	Env-CoP2; W18 vs. W36	Yes	0.0211	Two-tailed paired t test
Fig. 4d	ConC capture ELISA	Env-CoP2; W34 vs. W36	Yes	<0.0001	Two-tailed paired t test

Supplementary table 1:
Statistical testing outputs for group comparisons in corresponding indicated panels from figure 2, figure 3, figure 4 and supplementary figure 3. VNA: Virus neutralization assay; GMT: geometric mean titer.

Supplementary Table 2

Group	Animal	MLV	ConS (1B; C)	TV1.21 (2, C)	Ce703010010_C4 (2; C)	ZM233 M.PB6 (2; C)	2759058_F10_B6 (2; C)	3728.v2.c6 (2; C)	Ce704810053_2B7 (2; C)	ZM215F. PB8 (2; C)	2969249 (2; C)	B005582-7_G7.8 (2; C)	25710-2.43 (2; C)	Ce1176_A3 (2; C)	Ce703010217_B6 (2; C)	246-F3_C10_2 (2; AC)	BJOX002000.03.2 (2; CRF07_BC)	CH119 (2; CRF07_BC)	398F1 (2; A)	CNE8 (2; CRF01_AE)	TRO.11 (2; B)	X2278 (2; B)	X1632.S2-B10 (2; G)	CNE55 (2; CRF01_AE)
Env- CoP1	1	<20	2,082	934	<20	<20	<20	58	110	<20	<20	<20	51	65	<20	<20	49	79	<20	<20	<20	<20	56	20
	2	<20	<20	156	<20	<20	<20	<20	36	<20	<20	<20	<20	21	<20	<20	38	<20	141	<20	<20	<20	25	20
	3	<20	1,561	1,364	<20	<20	<20	<20	42	21	64	<20	105	<20	<20	<20	37	<20	849	<20	<20	<20	128	<20
	4	<20	111	4,466	<20	<20	22	<20	43	24	<20	<20	33	41	<20	<20	48	40	94	<20	<20	<20	45	20
	5	<20	262	379	<20	24	44	<20	44	29	36	<20	<20	31	34	<20	200	43	1,296	20	48	101	54	20
	6	<20	99	271	<20	<20	35	26	22	<20	53	<20	<20	21	27	<20	185	21	102	<20	<20	<20	22	<20
	7	<20	4,147	4,348	<20	<20	25	70	44	<20	<20	<20	334	94	41	42	390	110	1,035	21	23	322	373	27
	8	<20	>4860	3,117	<20	<20	21	111	81	<20	<20	<20	<20	<20	<20	<20	321	95	<20	<20	<20	<20	406	20
	9	<20	<20	2,328	<20	<20	<20	<20	88	<20	<20	<20	<20	27	<20	<20	174	<20	<20	<20	28	23	25	<20
Env-CoP2	10	<20	556	4,225	<20	<20	<20	<20	<20	<20	<20	<20	<20	78	29	<20	<20	<20	2,024	<20	<20	40	337	<20
	11	<20	<20	2,763	<20	<20	53	240	1,504	<20	342	<20	<20	76	22	<20	339	257	2,586	<20	<20	33	192	21
	12	<20	890	417	<20	<20	<20	<20	<20	<20	<20	<20	288	<20	<20	<20	116	<20	<20	<20	<20	<20	464	22
	13	<20	>4860	3,879	<20	<20	<20	43	172	<20	<20	<20	<20	30	<20	<20	169	301	260	<20	<20	<20	99	20
	14	<20	>4860	>4860	<20	<20	173	<20	<20	<20	177	<20	31	107	<20	<20	194	61	<20	<20	25	201	161	<20
	15	<20	<20	>4860	<20	<20	<20	<20	<20	<20	<20	<20	24	43	<20	<20	192	<20	78	<20	<20	32	209	20
	16	<20	2,525	1,068	<20	<20	<20	<20	<20	<20	<20	<20	43	20	<20	<20	39	<20	<20	<20	<20	<20	<20	<20
	17	<20	>4860	651	<20	<20	30	<20	25	24	<20	<20	<20	25	20	<20	183	21	<20	<20	<20	68	127	20
	18	<20	<20	>4860	<20	<20	<20	<20	112	<20	<20	<20	39	25	<20	<20	259	<20	<20	<20	30	37	94	<20
Env-CoP3	19	<20	82	623	<20	24	26	<20	44	<20	82	<20	31	37	20	<20	110	<20	66	<20	<20	<20	117	<20
	20	<20	550	891	<20	<20	40	<20	<20	<20	<20	<20	<20	<20	<20	<20	252	<20	<20	<20	<20	<20	45	<20
	21	<20	>4860	539	<20	<20	43	<20	<20	<20	<20	<20	<20	<20	<20	<20	96	<20	<20	<20	<20	35	90	<20
	22	<20	<20	828	<20	<20	<20	<20	<20	<20	<20	<20	<20	<20	<20	<20	41	<20	<20	<20	<20	<20	22	<20
	23	<20	>4860	135	<20	<20	<20	<20	<20	<20	<20	<20	<20	<20	<20	<20	<20	<20	<20	<20	<20	<20	<20	<20
	24	<20	<20	561	<20	<20	<20	96	<20	<20	<20	<20	<20	32	<20	<20	218	487	<20	<20	<20	<20	<20	<20
	25	<20	101	201	<20	<20	<20	38	<20	<20	<20	<20	<20	<20	<20	<20	21	<20	<20	<20	<20	<20	<20	20
	26	<20	503	508	<20	<20	<20	<20	<20	<20	<20	<20	<20	<20	<20	<20	<20	<20	<20	<20	<20	<20	20	<20
Control	27	<20	<20	<20	<20	<20	<20	<20	<20	<20	<20	<20	<20	<20	<20	<20	<20	<20	<20	<20	<20	<20	<20	<20
	28	<20	<20	<20	<20	<20	<20	<20	<20	<20	<20	<20	<20	<20	<20	<20	<20	<20	<20	<20	<20	<20	<20	<20
	29	<20	<20	<20	<20	<20	<20	<20	<20	<20	<20	<20	<20	<20	<20	<20	<20	<20	<20	<20	<20	<20	<20	<20
	30	<20	<20	<20	<20	<20	<20	<20	<20	<20	<20	<20	<20	<20	<20	<20	<20	<20	<20	<20	<20	<20	<20	<20

Clade C panel (columns ConS (1B; C) through 2969249 (2; C)) — Global panel (columns B005582-7_G7.8 (2; C) through CNE55 (2; CRF01_AE))

Supplementary table 2:

Table shows raw serum neutralization ID50 values from week 18 sera depicted in figure 3a. Values above the limit of detection (serum ID50 of 20) are highlighted in grey. Numbers next to the groups denote animal ID. Source data are provided as a Source Data file. MLV; Murine leukemia virus.

Supplementary Table 3

Group	Animal	PGT145	PGDM1400	VRC26.25	PG16	PGT128	10-1074	2G12	PGT121	VRC01	N6	3BNC117	CH103	VRC34.01	ACS202	PGT151	8ANC195	35O22	10E8	4E10	2F5
		V1/V2 loop				V3 loop				CD4bs				Fusion peptide			Interface		MPER		
Env- CoP1	1	0.000	0.000	0.005	0.011	0.000	0.009	0.000	0.000	0.000	0.003	0.000	0.000	0.016	0.009	0.022	0.012	0.000	0.003	0.000	0.000
	2	0.000	0.000	0.000	0.000	0.000	0.007	0.000	0.000	0.000	0.007	0.000	0.005	0.012	0.000	0.018	0.007	0.000	0.000	0.000	0.008
	3	0.000	0.000	0.000	0.000	0.000	0.004	0.000	0.000	0.000	0.040	0.000	0.002	0.014	0.000	0.026	0.038	0.000	0.000	0.002	0.048
	4	0.000	0.000	0.000	0.000	0.000	0.015	0.001	0.000	0.000	0.004	0.000	0.010	0.022	0.000	0.034	0.006	0.000	0.000	0.000	0.003
	5	0.000	0.000	0.000	0.000	0.000	0.033	0.009	0.023	0.000	0.034	0.000	0.006	0.018	0.000	0.041	0.044	0.000	0.000	0.000	0.030
	6	0.000	0.000	0.000	0.000	0.000	0.016	0.002	0.005	0.000	0.003	0.000	0.008	0.017	0.000	0.027	0.005	0.000	0.000	0.000	0.000
	7	0.000	0.000	0.000	0.000	0.000	0.026	0.019	0.001	0.000	0.049	0.000	0.015	0.037	0.000	0.087	0.103	0.000	0.012	0.000	0.054
	8	0.000	0.000	0.023	0.000	0.000	0.000	0.000	0.000	0.000	0.001	0.000	0.006	0.009	0.000	0.024	0.048	0.000	0.018	0.000	0.000
	9	0.000	0.001	0.000	0.000	0.000	0.006	0.006	0.000	0.000	0.002	0.000	0.004	0.006	0.000	0.017	0.019	0.000	0.004	0.000	0.000
Env-CoP2	10	0.000	0.000	0.000	0.000	0.000	0.000	0.004	0.000	0.023	0.037	0.000	0.011	0.014	0.000	0.043	0.046	0.000	0.000	0.000	0.039
	11	0.000	0.000	0.000	0.000	0.000	0.037	0.004	0.000	0.000	0.022	0.000	0.025	0.056	0.000	0.089	0.029	0.000	0.000	0.000	0.023
	12	0.041	0.000	0.030	0.000	0.000	0.000	0.000	0.000	0.000	0.000	0.000	0.019	0.009	0.000	0.000	0.017	0.000	0.007	0.000	0.004
	13	0.000	0.000	0.000	0.000	0.000	0.027	0.003	0.000	0.000	0.012	0.000	0.005	0.040	0.034	0.053	0.015	0.000	0.000	0.000	0.006
	14	0.000	0.000	0.000	0.019	0.000	0.003	0.026	0.000	0.000	0.008	0.000	0.000	0.006	0.000	0.037	0.078	0.017	0.020	0.000	0.000
	15	0.000	0.000	0.000	0.000	0.000	0.007	0.007	0.000	0.000	0.023	0.000	0.004	0.012	0.000	0.031	0.042	0.000	0.004	0.000	0.026
	16	0.005	0.000	0.004	0.000	0.003	0.002	0.000	0.003	0.000	0.000	0.000	0.002	0.002	0.000	0.000	0.000	0.000	0.000	0.000	0.000
	17	0.000	0.000	0.010	0.000	0.000	0.000	0.010	0.000	0.000	0.003	0.000	0.003	0.001	0.000	0.020	0.057	0.000	0.017	0.000	0.000
	18	0.000	0.001	0.011	0.000	0.000	0.000	0.009	0.000	0.000	0.003	0.000	0.003	0.000	0.000	0.019	0.055	0.000	0.017	0.000	0.000
Env-CoP3	19	0.000	0.000	0.002	0.000	0.000	0.008	0.001	0.000	0.000	0.017	0.000	0.005	0.015	0.000	0.026	0.020	0.000	0.000	0.000	0.021
	20	0.000	0.000	0.010	0.000	0.000	0.001	0.000	0.000	0.000	0.001	0.000	0.004	0.006	0.000	0.013	0.021	0.000	0.008	0.000	0.000
	21	0.000	0.001	0.008	0.000	0.000	0.000	0.005	0.000	0.000	0.002	0.000	0.002	0.001	0.000	0.012	0.033	0.000	0.010	0.000	0.000
	22	0.000	0.000	0.002	0.000	0.000	0.001	0.000	0.000	0.000	0.000	0.000	0.001	0.002	0.000	0.004	0.003	0.000	0.001	0.000	0.000
	23	0.000	0.000	0.000	0.000	0.000	0.000	0.000	0.000	0.000	0.000	0.000	0.000	0.000	0.000	0.000	0.000	0.000	0.000	0.000	0.000
	24	0.000	0.000	0.000	0.000	0.000	0.021	0.000	0.000	0.000	0.007	0.000	0.000	0.034	0.041	0.037	0.003	0.000	0.000	0.001	0.000
	25	0.000	0.000	0.000	0.000	0.000	0.000	0.000	0.000	0.000	0.000	0.000	0.000	0.000	0.000	0.000	0.000	0.000	0.000	0.000	0.000
	26	0.000	0.000	0.000	0.000	0.000	0.000	0.000	0.000	0.000	0.000	0.000	0.000	0.000	0.000	0.000	0.000	0.000	0.000	0.000	0.000

Supplementary table 3:
Table shows raw prediction score values from the neutralization fingerprinting results depicted in figure 3c. Values above 0.01 are highlighted in grey. Numbers next to the groups denote animal ID. Source data are provided as a Source Data file. CD4bs: CD4 binding site; MPER: membrane proximal external region.
