# Supplementary material for: CoPoP liposomes displaying stabilized clade C HIV-1 Env elicit tier 2 multiclade neutralization in rabbits
Source: Nat Commun. 2024 Apr 11;15:3128. doi: 10.1038/s41467-024-47492-1 (PMC11009251; doi:10.1038/s41467-024-47492-1)
Supplement: Supplementary file 3 — Reporting Summary [file 41467_2024_47492_MOESM3_ESM.pdf]

nature portfolio

Corresponding author(s): Frank Wegmann and Johannes P. M. Langedijk

Last updated by author(s): Mar 22, 2024

Reporting Summary

Nature Portfolio wishes to improve the reproducibility of the work that we publish. This form provides structure for consistency and transparency in reporting. For further information on Nature Portfolio policies, see our Editorial Policies and the Editorial Policy Checklist.

Statistics

For all statistical analyses, confirm that the following items are present in the figure legend, table legend, main text, or Methods section.

n/a	Confirmed	
☐	☒	The exact sample size (*n*) for each experimental group/condition, given as a discrete number and unit of measurement
☐	☒	A statement on whether measurements were taken from distinct samples or whether the same sample was measured repeatedly
☐	☒	The statistical test(s) used AND whether they are one- or two-sided<br>*Only common tests should be described solely by name; describe more complex techniques in the Methods section.*
☐	☒	A description of all covariates tested
☐	☒	A description of any assumptions or corrections, such as tests of normality and adjustment for multiple comparisons
☐	☒	A full description of the statistical parameters including central tendency (e.g. means) or other basic estimates (e.g. regression coefficient) AND variation (e.g. standard deviation) or associated estimates of uncertainty (e.g. confidence intervals)
☐	☒	For null hypothesis testing, the test statistic (e.g. *F*, *t*, *r*) with confidence intervals, effect sizes, degrees of freedom and *P* value noted<br>*Give P values as exact values whenever suitable.*
☒	☐	For Bayesian analysis, information on the choice of priors and Markov chain Monte Carlo settings
☒	☐	For hierarchical and complex designs, identification of the appropriate level for tests and full reporting of outcomes
☒	☐	Estimates of effect sizes (e.g. Cohen's *d*, Pearson's *r*), indicating how they were calculated

Our web collection on statistics for biologists contains articles on many of the points above.

Software and code

Policy information about availability of computer code

Data collection	HIV-1 Env specific and His-Tag specific ELISA luminescence readout was obtained in the Gen5™ Data Analysis Software.<br>Flow cytometry data was collected with BD FACSDiva software v.9.0.<br>DLS and nanoDSF data was collected with Uncle Client software v.5.03 (Unchained Labs).<br>AlphaLISA data was collected with PerkinElmer Kaleido software version 3.0.3067.117.
Data analysis	Gen5™ Data Analysis Software for calculating HIV-1 Env specific ELISA titers.<br>His-Tag specific ELISA titers were calculated with an in-house R-script.<br>Graph Pad Prism versions 9.0.0 and 9.5.0 for plotting graphs.<br>FlowJo software v.10.7.1 was used for flow cytometry data analysis.<br>Uncle Client software v.5.03 was used for calculation of melting temperatures.<br>CLC Main Workbench version 23.0.4 for sequence analysis.<br>R version 4.0.2 with the lsei package for neuralization fingerprinting data analysis.

For manuscripts utilizing custom algorithms or software that are central to the research but not yet described in published literature, software must be made available to editors and reviewers. We strongly encourage code deposition in a community repository (e.g. GitHub). See the Nature Portfolio guidelines for submitting code & software for further information.

Data

Policy information about availability of data

All manuscripts must include a data availability statement. This statement should provide the following information, where applicable:

- Accession codes, unique identifiers, or web links for publicly available datasets
- A description of any restrictions on data availability
- For clinical datasets or third party data, please ensure that the statement adheres to our policy

All of the final data has been included in main figures or supplementary information. Any requests for protocols and reagents should be directed to the corresponding authors to be fulfilled under reasonable request. Source data are provided with this paper.

Research involving human participants, their data, or biological material

Policy information about studies with human participants or human data. See also policy information about sex, gender (identity/presentation), and sexual orientation and race, ethnicity and racism.

Reporting on sex and gender	Not Applicable
Reporting on race, ethnicity, or other socially relevant groupings	Not Applicable
Population characteristics	Not Applicable
Recruitment	Not Applicable
Ethics oversight	Not Applicable

Note that full information on the approval of the study protocol must also be provided in the manuscript.

Field-specific reporting

Please select the one below that is the best fit for your research. If you are not sure, read the appropriate sections before making your selection.

☒ Life sciences ☐ Behavioural & social sciences ☐ Ecological, evolutionary & environmental sciences

For a reference copy of the document with all sections, see nature.com/documents/nr-reporting-summary-flat.pdf

Life sciences study design

All studies must disclose on these points even when the disclosure is negative.

Sample size	The group sample size of 9 was selected to provide enough statistical power to assess differences in VNA breadth.
Data exclusions	All data is included and shown.
Replication	ELISA and neutralization serum samples were always measured as technical duplicates. Octet (fig 1a) has been performed as technical replicates from a single experiment. DSF (fig 1b) was performed as a single measurement. DLS (fig 1c) has been performed as technical replicates and representative dataset was selected from four independent experiments. Cryo-EM (fig 1d) has been performed as a single experiment. Representative AlphaLISA dataset (fig 1e) was selected from three independent experiments. AlphaLISA (fig 1f) was performed as technical replicates and has been performed as a single experiment. FACS was performed as technical replicates and representative dataset was selected from four independent experiments. All attempts at replication were successful.
Randomization	Rabbits were randomly allocated into groups. Previous studies showed minimal differences in immunogenicity between genders and hence to keep all groups consistent, only female rabbits were used.
Blinding	Investigators were not blinded during ELISA and VNA data collection, but serum samples were randomly mixed onto the measurement plates for ELISA measurements to minimizing group specific biases in data collection. Investigators were blinded during cryo-EM analysis. Investigators were not blinded during Octet, DSF, DLS, AlphaLISA and FACS experiments, since there was no preference towards use of any of the three vaccine formulations and hence there was no scope for bias in unblinded testing.

Reporting for specific materials, systems and methods

We require information from authors about some types of materials, experimental systems and methods used in many studies. Here, indicate whether each material, system or method listed is relevant to your study. If you are not sure if a list item applies to your research, read the appropriate section before selecting a response.

Materials & experimental systems

n/a	Involved in the study	
☐	☒	Antibodies
☐	☒	Eukaryotic cell lines
☒	☐	Palaeontology and archaeology
☐	☒	Animals and other organisms
☒	☐	Clinical data
☒	☐	Dual use research of concern
☒	☐	Plants

Methods

n/a	Involved in the study	
☒	☐	ChIP-seq
☐	☒	Flow cytometry
☒	☐	MRI-based neuroimaging

Antibodies

Antibodies used

PGT145, PGDM1400, VRC26, PGT128, b6, F105, 17b, CR9506, 3BNC60, VRC34, PGT151 and 35O22 were produced in-house Janssen.
447-52D is from Polymun Scientific product no. AB014.
2G12: Polymun Scientific product no. AB002.
14e (provided by J.E. Robinson).
Clone numbers not applicable for the monoclonals above, source references included below.
Polyclonal Goat anti-human IgG Alexa fluor 647 is from Invitrogen catalog no. A-21445.
Polyclonal Goat anti-rabbit IgG HRP is from Jackson ImmunoResearch catalog no. 111-035-046.
Polyclonal Mouse anti-human IgG HRP is from Jackson ImmunoResearch catalog no. 209-035-098.

Validation

PGT145 and PGT128 were originally validated in DOI: 10.1038/nature10373
PGDM1400 was validated in doi:10.1073/pnas.1415789111
VRC26 was validated in doi:10.1038/nature13036
b6 was validated in doi:10.1128/jvi.77.1.642-658.2003
F105 was validated in doi:10.1128/JVI.79.20.13060-13069.2005
17b was validated in doi:10.1021/bi001397m
2G12 was validated in doi:10.1128/JVI.70.2.1100-1108.1996
14 was validated in doi:10.1016/j.virol.2013.10.007
447-52D was originally validated in DOI: 10.1128/JVI.66.12.7538-7542.1992
CR9506 was orginally validated in DOI: 10.1086/514115
3BNC60 was validated in doi:10.1126/science.1207227
VRC34 was validated in doi:10.1126/science.aae0474
PGT151 was validated in doi:10.1016/j.immuni.2014.04.009
35O22 was validated in doi:10.1038/nature13601
Goat anti-rabbit IgG HRP and Mouse anti-human IgG HRP were validated by immunoelectrophoresis and/or ELISA to react with the Fc portions of rabbit or human IgG heavy chain respectively by supplier.
Goat anti-human IgG AF647 was validated by the supplier by relative expression to ensure that the antibody binds to the antigen stated.

Eukaryotic cell lines

Policy information about cell lines and Sex and Gender in Research

Cell line source(s)

TZM-bl cells were sourced from NIH-ARP (female origin) (ARP-8129).
Human embryonic kidney cell line HEK293E-253 (female origin) (ATCC CRL 10852)
Lung carcinoma epithelial cells A549 (male origin) (BioWhittaker 71-217F)
PERC.6® TetR human embryonic retina cells immortalized by E1 to which a nucleotide encoding TetR has been introduced (sex unspecified) (in-house preparation Janssen Vaccines & Prevention, doi:10.1089/hum.1998.9.13-1909 (1998))

Authentication

PERC.6® TetR was authenticated in-house at Janssen Vaccines & Prevention.
The other cell lines were not formally authenticated by the authors.
TZM-bl is a HeLa cell line previously described to express luciferase and β-galactosidase genes under control of the human immunodeficiency virus type 1 (HIV-1) promoter by supplier.

Mycoplasma contamination

TZM-bl, HEK293E, A549, and PERC6® TetR cells tested as mycoplasma negative by suppliers.

Commonly misidentified lines
(See ICLAC register)

TZM-bl cells were previously described as JC-53-bl (clone 13).

Animals and other research organisms

Policy information about studies involving animals; ARRIVE guidelines recommended for reporting animal research, and Sex and Gender in Research

Laboratory animals

16-18 weeks old (at first immunization) female New Zealand White rabbits were used.

Wild animals	No wild animals were used in the study
Reporting on sex	Adult female rabbits used in the study.
Field-collected samples	No field-collected samples were used in the study.
Ethics oversight	Study performed in compliance with the U.S. Department of Agriculture's (USDA) Animal Welfare Act (9 CFR Parts 1, 2, and 3); the Guide for the Care and Use of Laboratory Animals (Institute of Laboratory Animal Resources, National Academy Press, Washington, D.C., 2011); and the National Institutes of Health, Office of Laboratory Animal Welfare. Animal work was approved by the Labcorp Early Development Laboratories, Denver Site Institutional Animal Care and Use Committee (IACUC) review board.

Note that full information on the approval of the study protocol must also be provided in the manuscript.

Flow Cytometry

Plots

Confirm that:

☒ The axis labels state the marker and fluorochrome used (e.g. CD4-FITC).

☒ The axis scales are clearly visible. Include numbers along axes only for bottom left plot of group (a 'group' is an analysis of identical markers).

☒ All plots are contour plots with outliers or pseudocolor plots.

☒ A numerical value for number of cells or percentage (with statistics) is provided.

Methodology

Sample preparation	A549 cells were transduced, harvested, stained and fixated as described in methods section
Instrument	FACS Canto instrument (BD Biosciences)
Software	BD FACSDiva v.9.0 (BD Biosciences) for collection and FlowJo v10.7.1 (Becton, Dickinson and Company) for analysis
Cell population abundance	a minimum of 23.000 cells for the final subpopulation (single, live cells) were collected
Gating strategy	Data were plotted as the median fluorescence intensity (APC channel) of the A549 single, live cell population (A549>single> live>MFI)

☒ Tick this box to confirm that a figure exemplifying the gating strategy is provided in the Supplementary Information.
